# From Fear of Missing Out to Poor Sleep: A Dual-Pathway Mechanism and the Selective Role of Sensation Seeking

**DOI:** 10.3390/bs16060920

**Published:** 2026-06-04

**Authors:** Yuantian Tong, Qinglu Xiao, Xiaojun Sun

**Affiliations:** 1 Mental Health Education Center, Zhejiang University of Technology, Hangzhou 310011, China; tyt2015@zjut.edu.cn; 2 Key Laboratory of Adolescent Cyberpsychology and Behavior (CCNU), Ministry of Education, School of Psychology, Central China Normal University, Wuhan 430079, China; xqlpsy0927@163.com

**Keywords:** Fear of Missing Out, sleep quality, problematic smartphone use, sensation seeking, dual-pathway model, behavioral amplification

## Abstract

Fear of Missing Out (FoMO) has been increasingly recognized as a psychological driver of problematic mobile phone use and sleep disturbances among young adults. However, existing research is fragmented, with limited integration of cognitive–affective and behavioral mechanisms within a unified theoretical framework. Drawing on the Interaction of Person-Affect-Cognition-Execution (I-PACE) model, a process-oriented framework in which problematic smartphone use mediates the association between FoMO and sleep quality, with sensation seeking examined as a boundary condition. Data from 1124 Chinese undergraduate students showed that FoMO was associated with poor sleep quality. Problematic smartphone use partially mediated this association, suggesting that FoMO is linked to sleep outcomes through both direct cognitive–affective processes and indirect behavioral pathways. Sensation seeking significantly strengthened the associations between FoMO and problematic smartphone use, as well as between problematic smartphone use and sleep quality, whereas it was not significantly associated with the direct FoMO-sleep link, indicating pathway-specific moderation primarily operating at the behavioral execution level. These findings provide empirical support for an I-PACE-based process model of FoMO-related sleep problems and highlight behavioral engagement processes as a key target for interventions among high-risk individuals.

## 1. Introduction

Sleep is a fundamental physiological need and a critical determinant of physical health, psychological well-being, and daily functioning (Gomez Fonseca & Genzel, 2020; Palmer et al., 2024). Adequate sleep supports immune restoration and cardiovascular functioning, whereas insufficient or poor-quality sleep has been consistently linked to a range of adverse outcomes, including heightened levels of depression, anxiety, and stress (W. Hu et al., 2026; J. Zhang et al., 2026). In recent years, growing attention has been directed toward digital media use as a potential risk factor for sleep disturbances, particularly among young adults, among whom sleep problems remain highly prevalent. Existing evidence suggests that approximately one-third of students worldwide experience varying degrees of sleep problems, with prevalence rates exceeding 40% in Chinese samples (Sivertsen et al., 2019; S. Zhang et al., 2023). This heightened vulnerability may reflect developmental characteristics of emerging adulthood, a period marked by increased autonomy, irregular routines, and heightened sensitivity to psychosocial stressors. Under conditions of academic pressure, social adjustment demands, and rapidly changing lifestyles, students’ sleep patterns are not only shaped by biological rhythms but are also particularly susceptible to behavioral and environmental influences (S. Zhang et al., 2023).

With the rapid development of mobile internet technologies, smartphones have become a central medium in the daily lives of university students, enabling continuous access to information and the maintenance of social connections (Shoval et al., 2020). This constant connectivity not only extends the duration of media use but also reshapes individuals’ temporal organization and attentional allocation, increasingly occupying the critical pre-sleep recovery window with information consumption and social interaction (Han et al., 2024). A growing body of research has documented a negative association between digital media use and sleep quality (Han et al., 2024). However, existing studies have largely focused on usage duration or frequency, offering limited insight into the underlying psychological mechanisms that drive persistent mobile phone use and explain individual differences in its impact on sleep. The present study adopts the Interaction of Person-Affect-Cognition-Execution (I-PACE) model as the overarching theoretical framework (Brand et al., 2016, 2019). This model emphasizes that problematic Internet-related behaviors emerge from the interaction among person-related characteristics, affective and cognitive responses, and executive control processes, which jointly contribute to the development and maintenance of maladaptive outcomes, including impairments in sleep. Based on this framework, the present study adopts a cognition–affect–behavior execution perspective to systematically examine how FoMO is related to sleep quality through problematic smartphone use, while further considering how individual personality differences (Sensation Seeking) shape the conditional strength of this process.

### 1.1. Fear of Missing Out and Sleep Quality

Within this context, Fear of Missing Out (FoMO), a key psychological driver in the digital age, has attracted increasing scholarly attention. FoMO refers to a persistent apprehension that others may be experiencing rewarding events in which one is absent (Przybylski et al., 2013). Beyond patterns of information use, it reflects an internally driven psychological state characterized by uncertainty regarding incomplete social information. FoMO is likely to be particularly salient among university students and readily reinforced by their developmental and contextual conditions. On the one hand, under increasing academic pressure, economic uncertainty, and intensified competition, students are more exposed to peer comparison and information about others’ achievements and opportunities. Perceiving oneself as lagging behind may heighten sensitivity to missing out (Hala, 2025). On the other hand, as a critical period of identity development, university life is marked by heightened sensitivity to evaluation and belonging, which further amplifies vigilance toward social information (J. Zhang et al., 2024). Under the combined influence of these individual characteristics and contextual factors, FoMO becomes particularly salient among university students and may adversely affect their sleep quality.

From the perspective of the I-PACE model, FoMO can be conceptualized as a cognitive–affective risk factor characterized by persistent concern about missing rewarding social experiences, heightened vigilance toward social information, and sustained cognitive–emotional activation. Such sustained cognitive–emotional activation may interfere with sleep initiation by maintaining elevated pre-sleep arousal. Specifically, FoMO-related cognition reflects a future-oriented and information-driven processing style, in which individuals remain focused on “potential but not yet accessed” social information. This unresolved cognitive state lacks a clear termination signal, thereby sustaining mental engagement even after external information input has ceased (Elhai et al., 2017). Because sleep initiation depends on the downregulation of cognitive arousal, such sustained activation may prolong sleep onset latency, reduce sleep efficiency, and ultimately impair sleep quality (Akbari et al., 2021). Therefore, FoMO, as a form of internally sustained cognitive activation, may be negatively associated with sleep quality. Accordingly, the present study proposes Hypothesis 1: Fear of Missing Out is significantly negatively associated with sleep quality among university students.

### 1.2. The Mediating Role of Problematic Smartphone Use

Although FoMO may be associated with sleep quality, its impact is not limited to the psychological domain but may also be amplified through behavioral pathways. From the perspective of the I-PACE model, FoMO-related arousal states formed at the cognitive–affective level may further drive individuals to engage in repetitive compensatory behaviors at the execution level in order to alleviate uncertainty and psychological discomfort (Brand et al., 2016, 2019). In the mobile internet environment, smartphones serve as the primary means through which individuals access information and maintain social connections, thereby becoming a direct channel for alleviating FoMO. Within this framework, the process reflects a transition from cognitive–affective responses to behavioral execution, whereby individuals use smartphones continuously to cope with uncertainty and anxiety triggered by FoMO. In FoMO-related contexts, concerns about missing information, social interactions, and developmental opportunities may be associated with greater information seeking and smartphone use, particularly browsing and social interaction behaviors (Elhai et al., 2021), thereby strengthening behavioral dependence. Moreover, the uncertainty and belonging-related anxiety underlying FoMO are rarely resolved through a single instance of information checking. Instead, individuals may engage in continuous and repetitive smartphone use to maintain a sense of informational control (Elhai et al., 2016; Putri et al., 2026). Over time, this pattern may shift from goal-directed use to a compensatory and habitual form of engagement, ultimately developing into problematic use that is difficult to regulate.

Existing research has demonstrated that problematic smartphone use is associated with poorer sleep quality, including delayed sleep onset, reduced sleep duration, and impaired sleep efficiency (Cemei et al., 2024). This relationship may be explained by multiple, interrelated mechanisms. First, from a time allocation perspective, problematic smartphone use may be associated with reduced time allocated to sleep, resulting in delayed bedtimes and reduced sleep duration (Yang et al., 2020). Second, from a psychological perspective, addictive use involves continuous information input and interactive engagement, which sustain elevated cognitive activation and hinder the relaxation necessary for sleep initiation (K. Wang & Scherr, 2022). Importantly, this sustained activation may persist after smartphone use has ceased, suggesting difficulties in post-use arousal downregulation. Furthermore, from a physiological standpoint, prolonged exposure to screen-emitted blue light before bedtime can suppress sleepiness and disrupt circadian rhythms (Heo et al., 2017). In addition, exposure to radio-frequency electromagnetic fields from mobile phones may alter sleep-related brain activity and electroencephalographic oscillations, potentially influencing sleep-related regulatory processes (Sousouri et al., 2025). Taken together, these temporal, psychological, and physiological mechanisms suggest that problematic smartphone use may function as a key behavioral pathway linking FoMO to sleep quality. Accordingly, the present study proposes Hypothesis 2: Problematic smartphone use mediates the relationship between FoMO and sleep quality among university students.

### 1.3. The Moderating Role of Sensation Seeking

Although the foregoing analysis outlines a general pathway linking FoMO, problematic smartphone use, and sleep quality, this process is unlikely to operate uniformly across individuals (Akbari et al., 2021). From the perspective of the I-PACE model, the development and maintenance of problematic Internet-related behaviors result from the interaction among person-related characteristics, affective and cognitive responses, and executive control processes (Brand et al., 2016, 2019). This implies that the same cognitive–affective experience may be expressed through different behavioral execution pathways across individuals. FoMO reflects a subjective experience of uncertainty related to missing social information, and its behavioral and psychological consequences depend not only on its intensity but also on individual differences in how such uncertainty is regulated and processed. Accordingly, identifying key personality traits that shape these responses is essential for understanding when and for whom FoMO is more likely to translate into maladaptive outcomes. In this context, sensation seeking provides an important individual-difference perspective for elucidating the boundary conditions of this process.

Sensation seeking is a personality trait characterized by the preference for varied, novel, complex, and intense stimuli, as well as the active pursuit of such experiences (Ye et al., 2014). According to Sensation Seeking Theory and Optimal Arousal Theory (Carrol et al., 1982), individuals high in sensation seeking are typically characterized by higher optimal arousal levels and a tendency to regulate arousal through engagement with external stimuli. In the context of FoMO, uncertainty related to missing social information may be more likely to be appraised by high sensation seekers as an opportunity for exploration and potential reward, thereby strengthening their tendency toward information seeking and continuous monitoring. From the perspective of Reinforcement Sensitivity Theory (Gray & McNaughton, 2000), digital social feedback such as likes and comments represents salient reward cues, and heightened reward sensitivity has been identified as a key predictor of problematic mobile phone use (Kwak & Kim, 2023). Taken together, sensation seeking may be associated with differences in how FoMO-related stimuli are appraised in terms of their novelty and reward value, and how such appraisals translate into behavioral responses, thereby facilitating repeated media engagement and reinforcing usage patterns. Consistent with prior evidence, individuals high in sensation seeking tend to exhibit stronger addictive tendencies (Tong et al., 2019). Accordingly, the present study proposes Hypothesis 3: Sensation seeking moderates the relationship between FoMO and problematic smartphone use among university students, such that this association is stronger among individuals high in sensation seeking.

Building on this, the role of sensation seeking may further extend to the outcome stage, particularly in the pathway linking problematic smartphone use to sleep quality. Previous research has shown that the impact of problematic smartphone use on sleep is not solely attributable to the behavior itself, but is also closely related to post-use recovery processes. Specifically, sleep initiation depends on an effective transition from a state of high arousal to low arousal, and this recovery process varies substantially across individuals (De Valck et al., 2004). From this perspective, individual differences in arousal downregulation may serve as an important boundary condition shaping the extent to which problematic smartphone use translates into impaired sleep quality. Sensation seeking represents a key trait relevant to this process. Rather than being limited to external stimulus seeking, sensation seeking also reflects individual differences in the regulation of internal activation states (Taylor & Hamilton, 1997). Individuals high in sensation seeking are typically characterized by a stronger tendency to maintain activation and relatively weaker behavioral disengagement and self-regulation capacities, which may in turn make it more difficult for them to downregulate arousal following smartphone use, leading to sustained pre-sleep activation and a less efficient transition into a low-arousal state (Riemann et al., 2010; Chen et al., 2021). Consequently, at comparable levels of problematic smartphone use, individuals high in sensation seeking may experience more pronounced sleep disturbances, reflecting their reduced efficiency in post-use recovery processes. Accordingly, the present study proposes Hypothesis 4: Sensation seeking moderates the relationship between problematic smartphone use and sleep quality, such that this association is stronger among individuals high in sensation seeking.

Furthermore, sensation seeking may also moderate the relationship between FoMO and sleep quality. According to the I-PACE model, person-related characteristics are associated not only with behavioral execution processes, but also with differences in information processing at the cognitive–affective stage, which may in turn be related to variations in functional outcomes. The association between FoMO and sleep quality may be linked to sustained cognitive and emotional activation, and the strength of this association may differ depending on individual attentional characteristics. From an information-processing perspective, FoMO is associated with a stable attentional orientation toward potential social information (Przybylski et al., 2013). However, individuals differ substantially in their tendency to maintain attention under conditions of uncertainty. Individuals high in sensation seeking may exhibit greater attentional engagement with and sustained focus on FoMO-related social cues, which may be associated with higher levels of cognitive and emotional arousal and poorer sleep quality (Taghvaee & Mazandarani, 2022). Accordingly, even at comparable levels of FoMO, individuals high in sensation seeking may be more likely to maintain attentional focus on FoMO-related information, which may strengthen the negative association between FoMO and sleep quality. Therefore, the present study proposes Hypothesis 5: sensation seeking moderates the relationship between FoMO and sleep quality among university students.

### 1.4. The Present Study

**Figure 1:**
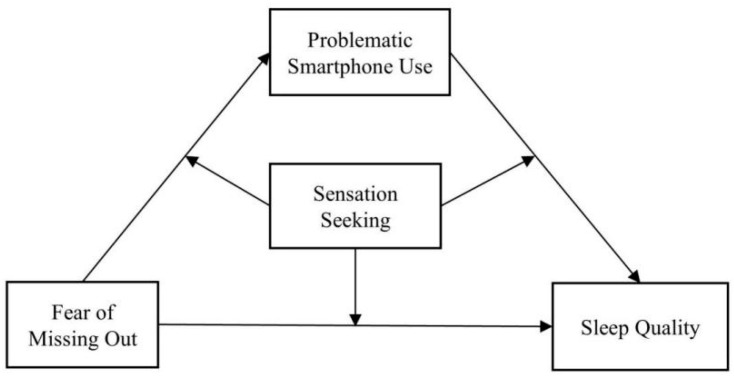
The proposed moderated mediation model.

To address the above research gap, the present study draws on the I-PACE model and an arousal regulation perspective to construct a moderated mediation model for examining the relationship between FoMO and sleep quality among university students. The proposed model emphasizes the interaction among person-related characteristics, cognitive–affective responses, and behavioral execution processes, and seeks to explain how FoMO is related to sleep quality through problematic smartphone use, as well as how this process is further shaped by individual difference factors. Specifically, the present study proposes that FoMO may be associated with poorer sleep quality both directly and indirectly through problematic smartphone use, while sensation seeking may condition these associations by shaping individual responses to FoMO-related uncertainty, behavioral engagement, and post-use recovery processes. Accordingly, the present study tests the following hypotheses: (1) FoMO is positively associated with poor sleep quality among university students; (2) problematic smartphone use mediates the relationship between FoMO and sleep quality; (3) sensation seeking moderates the relationship between FoMO and problematic smartphone use; (4) sensation seeking moderates the relationship between problematic smartphone use and sleep quality; and (5) sensation seeking moderates the direct relationship between FoMO and sleep quality. By examining these pathways simultaneously, this study aims to clarify how and under what conditions FoMO is associated with sleep disturbance in the digital context. The proposed moderated mediation model is depicted in Figure 1.

## 2. Materials and Methods

### 2.1. Ethical Statement and Informed Consent

Ethical approval for this study was provided by the Institutional Review Board at Central China Normal University (Approval No. CCNU-IRB-202406034b). The study was conducted in accordance with the ethical standards of the institutional research committee and the Declaration of Helsinki. All participants provided written informed consent prior to participation. Participation was voluntary, and all responses were collected anonymously to ensure confidentiality. No identifying information was collected during the survey.

### 2.2. Participants and Procedure

In this study, a convenience sampling method was adopted to recruit first-year, second-year, and third-year undergraduate students from two full-time universities in Hangzhou and Wuhan, China. Fourth-year students were not included in the questionnaire distribution, as they were primarily occupied with job hunting and internships. Data collection was conducted during general education courses for undergraduate students, including Mental Health Education for College Students, College English, Fundamentals of Computer Science, and other interdisciplinary elective courses. These courses are designed to be accessible to students from multiple academic disciplines across the universities, which facilitated the recruitment of a relatively diverse sample. All questionnaires were administered in Chinese to ensure linguistic consistency and cultural appropriateness for the participants. The surveys were administered in classrooms by professionally trained teachers, with participants being informed that participation was voluntary and anonymous, and they should choose the option that best represented their feelings. Participants were given 30 min to complete the questionnaire, and the average time to complete all questions was 20 min. A total of 1124 data were included in the study, of whom 498 (44.3%) were boys and 626 (55.7%) were girls; 329 (29.3%) were Grade 1 students, 432 (38.4%) were Grade 2 students; 363 (32.3%) were Grade 3 students. The average age of them was 19.65 years old (SD = 1.20).

### 2.3. Measurements

#### 2.3.1. Fear of Missing Out

Participants’ fear of missing out (FoMO) was assessed using the 10-item FoMO scale (Przybylski et al., 2013), rated on a 5-point Likert scale (from 1 “Not at all true of me” to 5 “Extremely true of me”). Higher total scores indicate high levels of FoMO. This scale has shown satisfactory reliability and validity in studies among Chinese students (Niu et al., 2022). In the present study, the Cronbach’s α for the scale was 0.71.

#### 2.3.2. Problematic Smartphone Use

Problematic smartphone use among university students was assessed using the Mobile Phone Addiction Index (MPAI) developed by Leung (2008) at the Chinese University of Hong Kong, a widely used instrument measuring manipulative patterns of mobile phone use. The scale consists of 17 items rated on a 5-point Likert scale (1 = never, 5 = always), with higher scores indicating a greater degree of problematic smartphone use. This scale has shown satisfactory reliability and validity in studies among Chinese students (Lian et al., 2018). In the current study, the Cronbach’s α for the scale was 0.86.

#### 2.3.3. Sensation Seeking

The Sensation Seeking Scale was revised by Steinberg et al. (2008) and subsequently translated into Chinese by Li et al. (2010). The scale consists of six items rated on a 6-point Likert scale (1 = strongly disagree, 6 = strongly agree), with higher scores indicating greater levels of sensation seeking. This scale has shown satisfactory reliability and validity in studies among Chinese students (Tong et al., 2019). In the present study, Cronbach’s α for the scale was 0.82.

#### 2.3.4. Sleep Quality

The Chinese version of the Pittsburgh Sleep Quality Index (PSQI), originally developed by Buysse et al. (1989) and later adapted by Liu et al. (1996), was employed to assess individuals’ sleep quality. The scale comprises 19 self-report items and 5 observer-rated items, with the 19th self-report item and all observer-rated items excluded from the scoring. These items are organized into seven components, each rated on a 0–3 scale. The component scores are summed to produce a global sleep quality index ranging from 0 to 21, where higher scores reflect poorer sleep quality.

### 2.4. Statistical Analyses

Descriptive statistics (means and standard deviations) and correlation analyses were conducted to examine the relationships among demographic variables, FoMO, problematic smartphone use, sensation seeking and sleep quality among college students. To further determine the predictive relationships among variables, the proposed moderated mediation model was tested using Hayes’ PROCESS macro for SPSS (Version 4.0; Model 4 and Model59; Hayes, 2013) in IBM SPSS Statistics (Version 27.0). Based on prior literature indicating that demographic characteristics, particularly gender and age, are commonly associated with both smartphone use behaviors and sleep-related outcomes (Amez et al., 2020; Demirci et al., 2015), these variables were included as control variables in subsequent analyses to account for potential confounding effects. Prior to moderation analyses, all continuous variables were mean-centered using the PROCESS macro to reduce multicollinearity between interaction terms. Significant interaction effects were further probed using simple slope analyses (Toothaker, 1994). Model assumptions were examined prior to hypothesis testing. Multicollinearity diagnostics indicated no serious multicollinearity concerns among the variables (all VIFs < 1.2). Residual histograms and normal probability (P-P) plots suggested that the residuals were approximately normally distributed and closely aligned with the diagonal line. No evidence of serious violations of linearity was observed.

## 3. Results

### 3.1. Descriptive Statistics and Correlation Analysis

The mean, standard deviation and correlation among all study variables are shown in Table 1. The results indicated that the key study variables were significantly intercorrelated. Consistent with the scale’s scoring direction, higher scores on the sleep quality scale (indicating poorer sleep quality) were significantly associated with FoMO, problematic smartphone use, and sensation seeking.

### 3.2. Testing for the Proposed Mediation Model

The mediating role of problematic smartphone use in the association between FoMO and sleep quality was examined using Model 4 of the PROCESS macro for SPSS (Hayes, 2013). As shown in Table 2 and Table 3, the total effect was significant (*β* = 0.27, *p* < 0.001), indicating that higher levels of FoMO were related to poorer sleep quality. After incorporating problematic smartphone use into the mediation model, FoMO significantly positively associated with higher problematic smartphone use (*β* = 0.38, *p* < 0.001), which in turn was positively associated with poorer sleep quality (*β* = 0.24, *p* < 0.001). The direct effect of FoMO on sleep quality was significant (*β* = 0.18, *p* < 0.001). Furthermore, the indirect effect of FoMO on sleep quality via problematic smartphone use was significant (*β* = 0.09, 95% CI = [0.06, 0.12]). Taken together, the PROCESS analyses revealed a significant indirect effect of FoMO on sleep quality through problematic smartphone use. Meanwhile, the direct effect of FoMO on sleep quality remained significant after problematic smartphone use was included in the model, indicating that the association was only partially accounted for by the behavioral pathway. These findings support Hypothesis 2. In addition, the significant total effect of FoMO on sleep quality provided support for Hypothesis 1.

### 3.3. Testing for the Proposed Moderated Mediation Model

The proposed moderated mediation model was tested using Model 59 of the PROCESS macro for SPSS (Hayes, 2013), in which sensation seeking was specified as a moderator of the first-stage (FoMO → problematic smartphone use), second-stage (problematic smartphone use → sleep quality), and direct (FoMO → sleep quality) pathways. 

As shown in Table 4, the interaction between FoMO and sensation seeking significantly associated with problematic smartphone use (*β* = 0.06, *p* < 0.05), indicating that sensation seeking moderated the association between FoMO and problematic smartphone use. In addition, the interaction between problematic smartphone use and sensation seeking was significantly associated with sleep quality (*β* = 0.06, *p* < 0.05), suggesting that sensation seeking also moderated the relationship between problematic smartphone use and sleep quality. Furthermore, the interaction between FoMO and sensation seeking was not significantly associated with sleep quality (*β* = 0.05, *p* > 0.05), indicating that sensation seeking did not significantly moderate the direct relationship between FoMO and sleep quality. These results support Hypotheses 3 and 4 and Hypothesis 5 was not supported.

**Figure 2:**
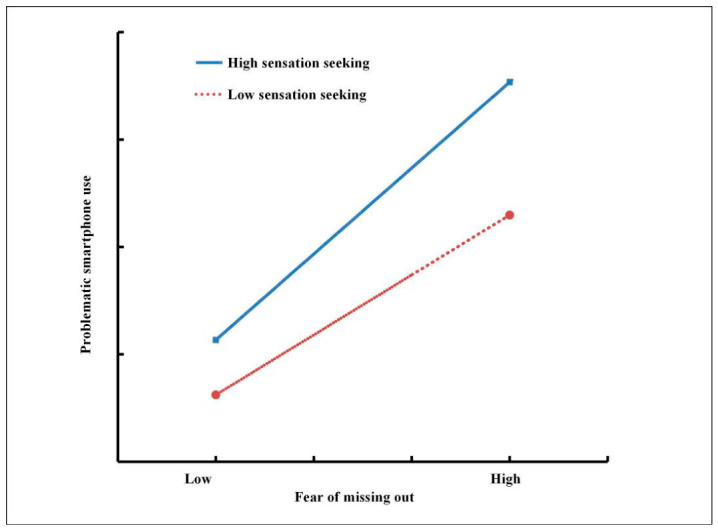
Sensation seeking as a moderator between fear of missing out and problematic smartphone use.

**Figure 3:**
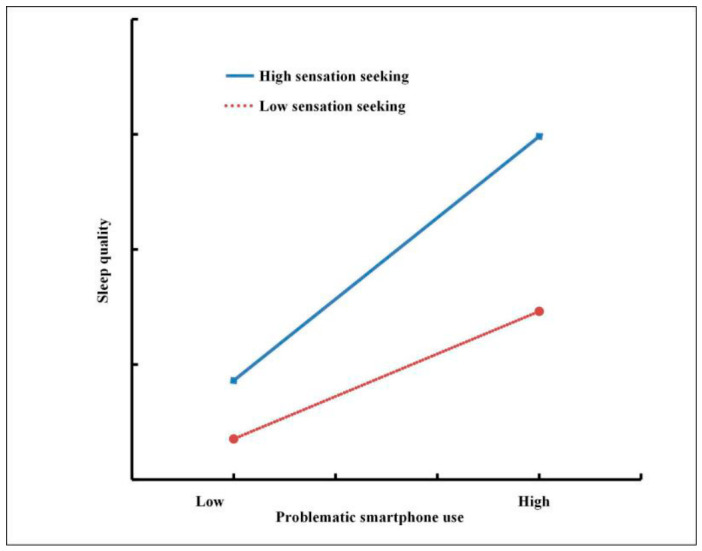
Sensation seeking as a moderator between problematic smartphone use and sleep quality.

Finally, the nature of the moderating effect was further elucidated by simple slope analysis. As shown in Table 5 and Figure 2, the effect of FoMO on problematic smartphone use was stronger among individuals with high levels of sensation seeking (*β* = 0.40, *t* = 12.72, *p* < 0.001) than among those with low levels of sensation seeking (*β* = 0.28, *t* = 6.12, *p* < 0.001). Similarly, as shown in Table 5 and Figure 3, the effect of problematic smartphone use on poor sleep quality was more pronounced among individuals with high sensation seeking (*β* = 0.27, *t* = 7.44, *p* < 0.001) compared to those with low sensation seeking (*β* = 0.16, *t* = 3.66, *p* < 0.001). These findings indicated that higher levels of sensation seeking could amplify both the effect of FoMO on problematic smartphone use and the effect of problematic smartphone use on sleep quality.

## 4. Discussion

Grounded in the Interaction of Person-Affect-Cognition-Execution (I-PACE) model (Brand et al., 2016, 2019), the present study provides an integrated explanation of how Fear of Missing Out (FoMO) is associated with sleep quality among college students through cognitive–affective processes, behavioral execution, and individual difference factors. Extending prior fragmented perspectives on problematic Internet use, the findings support a process-oriented pathway in which FoMO operates as a cognitive–affective vulnerability factor that may increase the likelihood of maladaptive smartphone use, which in turn is associated with impaired sleep quality. In addition, sensation seeking significantly moderated both the path from FoMO to problematic smartphone use and the path from problematic smartphone use to sleep quality, but did not moderate the direct association between FoMO and sleep quality. Taken together, these findings provide an integrated account of FoMO-related sleep problems within the I-PACE framework, highlighting the dynamic interplay among person-related characteristics (sensation seeking), cognitive–affective processes (FoMO), and behavioral execution (problematic smartphone use) in shaping sleep outcomes.

### 4.1. The Relationship Between FoMO and Sleep Quality

The present study found that FoMO significantly associated with sleep quality among college students, supporting Hypothesis 1 and aligning with prior research. From a motivational perspective, as suggested by Self-Determination Theory (Ryan & Deci, 2000), FoMO may reflect a state of persistent need-related tension, particularly with regard to relatedness. This unfulfilled state may contribute to sustained cognitive arousal at bedtime, thereby disrupting sleep initiation (Elhai et al., 2017). Within the I-PACE framework, this cognitive–affective state may be conceptualized as an early-stage vulnerability factor characterized by heightened sensitivity to social information and ongoing informational uncertainty (Brand et al., 2019). Unlike responses to specific and identifiable threats, FoMO involves a diffuse concern about potentially missing socially rewarding experiences or opportunities (Przybylski et al., 2013). Given the continuous and rapidly updating nature of social media environments, such uncertainty may lack a clear cognitive endpoint, making disengagement from online monitoring more difficult and potentially interfering with pre-sleep cognitive deactivation. In this sense, FoMO-related cognitive activation may be linked to prolonged sleep onset latency and reduced sleep quality (Akbari et al., 2021). Taken together, these findings suggest that cognitive–affective processes related to social uncertainty and persistent information monitoring may represent important mechanisms linking FoMO to sleep problems within the I-PACE framework. It is also noteworthy that the mean PSQI score observed in the present study was relatively high, indicating generally poor sleep quality among participants. This finding is broadly consistent with previous evidence suggesting that sleep disturbances are highly prevalent among university students, particularly among Chinese college students (Lund et al., 2010; S. Zhang et al., 2023). University students are often exposed to substantial academic demands, irregular schedules, and extensive digital media engagement, all of which have been identified as important correlates of sleep problems (J. Wang et al., 2025; L. Hu et al., 2024). In addition, within the contemporary Chinese higher-education context, study-related and information-seeking activities frequently extend into late-night hours, which may further disrupt sleep timing and reduce sleep opportunity. Therefore, the relatively elevated PSQI scores observed in the present study may reflect broader epidemiological and contextual characteristics of contemporary university student populations rather than an atypical sample characteristic.

### 4.2. The Mediating Role of Problematic Smartphone Use

The present study further demonstrated that problematic smartphone use partially mediated the association between FoMO and sleep quality, supporting Hypothesis 2. This finding is consistent with the Compensatory Internet Use Theory, which posits that individuals engage in digital media use to compensate for unmet psychological needs (Kardefelt-Winther, 2014). In the context of FoMO, heightened sensitivity to social uncertainty and concerns about missing rewarding experiences may be associated with more frequent smartphone checking and information-monitoring behaviors, as individuals attempt to obtain immediate social feedback and reduce uncertainty (Elhai et al., 2016; Putri et al., 2026). Importantly, this process may reflect a negative reinforcement pattern. Because online social information is continuously updated and inherently difficult to fully resolve, the reduction in uncertainty is often temporary. As a result, individuals may repeatedly return to smartphone-mediated information seeking, gradually forming more persistent and maladaptive usage tendencies over time. The present findings further suggest that problematic smartphone use may be linked to poorer sleep quality through several interrelated behavioral and psychophysiological pathways. First, prolonged smartphone engagement may delay bedtime and reduce available sleep time through behavioral displacement (Yang et al., 2020). Second, FoMO-related monitoring tendencies may maintain elevated cognitive arousal before sleep, particularly through continued anticipation of incoming information and social updates (K. Wang & Scherr, 2022). In addition, pre-sleep exposure to screen light, continuous mobile device engagement, and smartphone-related physiological stimulation may further interfere with sleep-related regulatory processes (Heo et al., 2017; Huiberts et al., 2022; Sousouri et al., 2025). Taken together, these findings support a process-oriented interpretation in which FoMO-related cognitive–affective activation is associated with maladaptive behavioral engagement, which may in turn be related to poorer sleep outcomes through the combined influence of time displacement, sustained cognitive arousal, and physiological interference.

### 4.3. The Moderating Role of Sensation Seeking

The present study found that sensation seeking demonstrated a pathway-specific moderating pattern within the proposed model. Specifically, sensation seeking significantly moderated the association between FoMO and problematic smartphone use (Hypothesis 3 supported), as well as the association between problematic smartphone use and sleep quality (Hypothesis 4 supported). However, it did not moderate the direct relationship between FoMO and sleep quality (Hypothesis 5 not supported). Collectively, these findings suggest that sensation seeking may be more closely associated with externally oriented behavioral engagement processes than with internally maintained cognitive–affective processes directly linked to FoMO.

First, sensation seeking moderated the association between FoMO and problematic smartphone use, such that the positive association between FoMO and problematic smartphone use was stronger among individuals with higher levels of sensation seeking. Within the I-PACE framework (Brand et al., 2019), sensation seeking can be conceptualized as a person-related characteristic that may influence how cognitive–affective vulnerabilities are translated into behavioral responses in digital environments. Consistent with optimal arousal theory and prior research (Carrol et al., 1982), individuals high in sensation seeking tend to prefer novelty, stimulation, and rapidly changing experiences. In the context of FoMO, the continuously updated and socially rewarding nature of smartphone-mediated information environments may be particularly attractive to these individuals. Accordingly, when experiencing FoMO-related uncertainty and concern about missing social information, individuals high in sensation seeking may be more inclined to engage in repeated smartphone checking and information-monitoring behaviors as a means of reducing uncertainty while simultaneously seeking stimulation from novel and rapidly changing information. This dual reinforcement mechanism—comprising uncertainty reduction and stimulation seeking—may increase the likelihood that individuals high in sensation seeking translate FoMO into addictive smartphone use behavior. Existing research has similarly shown that sensation seeking is positively associated with problematic smartphone use (Tong et al., 2019). In addition, from the perspective of reward sensitivity theories (Gray & McNaughton, 2000), individuals high in sensation seeking may exhibit heightened responsiveness to reward-related informational cues, which may further strengthen sustained engagement with smartphone-mediated social information.

Second, sensation seeking significantly moderated the association between problematic smartphone use and sleep quality, such that the negative association between problematic smartphone use and sleep quality was stronger among individuals with high sensation seeking. The present findings suggest that sensation seeking may be particularly relevant during the post-engagement recovery stage of digital media use. Individuals high in sensation seeking tend to exhibit relatively elevated arousal tendencies and stronger orientation toward continued stimulation (Ye et al., 2014), which may make disengagement from smartphone-related stimulation more difficult during the pre-sleep period. In addition, residual cognitive activation associated with continued information anticipation and mental engagement may further maintain elevated pre-sleep arousal (Riemann et al., 2010). Within the I-PACE framework (Brand et al., 2019), this pattern may reflect prolonged interaction between behavioral execution processes and affective-physiological regulation systems. Thus, the association between problematic smartphone use and poorer sleep quality may become more pronounced among individuals high in sensation seeking due to greater difficulty in arousal down-regulation following smartphone engagement.

In contrast, sensation seeking did not significantly moderate the direct association between FoMO and sleep quality (H5 not supported). Despite this null finding, it may still be theoretically informative. Specifically, the direct association between FoMO and sleep quality may primarily reflect internally maintained cognitive–affective processes, such as rumination, anticipatory thinking, and persistent cognitive activation related to potentially missed social experiences (Flores et al., 2023). These processes are relatively independent of external stimulus engagement, which may explain why sensation seeking—more strongly linked to approach tendencies toward external stimulation and behavioral activation—shows limited relevance for this direct pathway. By contrast, sensation seeking significantly moderated the indirect pathways involving problematic smartphone use, which involved sustained behavioral engagement with mobile devices and repeated exposure to continuously updated digital information. Individuals high in sensation seeking may be more responsive to such stimulation-rich environments (Ye et al., 2014), and thus may be more likely to engage in persistent information-monitoring and smartphone-checking behaviors when experiencing FoMO-related uncertainty.

Taken together, the present findings suggest that the association between FoMO and sleep quality may vary in its sensitivity to personality traits depending on the underlying pathway. In particular, the direct cognitive–affective pathway appears to be relatively less contingent on individual differences in sensation seeking, whereas the indirect behavioral pathway involving problematic smartphone use appears more responsive to such traits. Within the I-PACE framework (Brand et al., 2016, 2019), this pattern may be interpreted as suggesting that cognitive–affective vulnerabilities such as FoMO are not equally associated with downstream functional outcomes across individuals, and that their associations with sleep-related outcomes may operate more strongly through behavioral execution processes.

### 4.4. Theoretical Contributions and Practical Implications

Based on the above results, this study not only validates the dual-pathway mechanism through which FoMO affects sleep, but also further reveals the selective moderating role of sensation seeking across different pathways, thereby addressing the key research questions proposed in the introduction. By integrating the Interaction of Person-Affect-Cognition-Execution (I-PACE) model with complementary perspectives on motivational and arousal-related processes, this study develops a moderated mediation framework that helps to specify when and how FoMO is linked to sleep-related outcomes in the digital context. In terms of theoretical contributions, this study makes three main contributions. First, it extends prior research by empirically supporting a sequential process in which FoMO, as a cognitive–affective vulnerability factor, is associated with problematic smartphone use, which in turn relates to sleep quality. This provides a more process-oriented account of digital media-related sleep problems. Second, by examining the moderating role of sensation seeking across different stages of the model, the findings reveal a pathway-specific pattern, suggesting that personality traits may not exert uniform effects across the entire FoMO-sleep process. Third, by distinguishing between a relatively internally oriented cognitive–affective pathway and a behaviorally driven execution pathway, the results indicate that sensation seeking is more relevant for behavioral engagement processes than for directly cognitive–affective associations with sleep outcomes, thereby refining the role of personality within the I-PACE framework.

At the practical level, these findings suggest that interventions for sleep problems among college students should be multi-level and mechanism-targeted. First, FoMO may be considered a relevant cognitive–affective risk factor in digital contexts, and interventions could focus on reducing uncertainty-related cognitive preoccupation and enhancing emotional regulation strategies. Second, given the mediating role of problematic smartphone use, intervention efforts should focus on reducing maladaptive compensatory use patterns and promoting healthier digital behavior regulation, particularly in the pre-sleep period. Strategies such as establishing structured “digital curfews” and encouraging alternative relaxation routines may help break the cycle of nighttime smartphone dependence. Third, for individuals high in sensation seeking, personalized interventions are warranted. Rather than attempting to suppress their need for stimulation, interventions should emphasize adaptive substitution strategies, such as channeling stimulation seeking into offline or physically engaging activities, combined with pre-sleep arousal-downregulation training to improve recovery efficiency.

### 4.5. Limitations and Future Directions

Although this study provides empirical support for an integrated theoretical model and its underlying mechanisms, several limitations should be acknowledged. First, the cross-sectional design limits causal inference. Future research is encouraged to adopt longitudinal or experience sampling methodologies to better capture the dynamic and temporal relationships among FoMO, problematic smartphone use, and sleep quality, thereby strengthening causal interpretations. Second, the sample was limited to Chinese college students, which may constrain the generalizability of the findings. As FoMO is closely embedded in social media use and social comparison processes, its expression and effects may vary across cultural contexts. Future studies should examine whether cultural values, social connectedness norms, and media usage patterns moderate the observed relationships, particularly in collectivistic versus individualistic societies. Third, although gender and age were included as control variables, other factors potentially associated with sleep quality, such as anxiety, depression, chronotype, physical activity, academic workload, and living environment, were not assessed in the present study. Therefore, the observed associations should be interpreted with caution, as the potential influence of unmeasured confounding variables cannot be fully excluded. Fourth, all key variables were assessed using self-report measures. Future research could integrate objective indicators to improve measurement validity. For example, wearable devices (e.g., actigraphy or smart bands) may be used to assess sleep parameters more precisely, while digital trace data (e.g., screen time and app usage logs) may complement self-reported smartphone use. Finally, the moderating role of sensation seeking warrants further mechanistic exploration. Future studies may adopt experimental paradigms or neuroscientific approaches to clarify the underlying processes. In particular, neuroimaging techniques such as EEG or fMRI could be employed to examine neural responses to FoMO-related stimuli among high sensation seekers, and to investigate how these neural patterns are associated with subsequent smartphone use behavior and sleep outcomes.

## 5. Conclusions

Grounded in the Interaction of Person–Affect–Cognition–Execution (I-PACE) model, this study examined a moderated mediation model to clarify the mechanisms through which Fear of Missing Out (FoMO) is associated with sleep quality among college students. The results indicated that FoMO was associated with poorer sleep quality both directly and indirectly through problematic smartphone use. In addition, sensation seeking demonstrated a pathway-specific moderating pattern. It strengthened the associations between FoMO and problematic smartphone use, as well as between problematic smartphone use and sleep quality, whereas it was not significantly associated with the direct FoMO-sleep relationship. This pattern suggests that personality traits may play a more prominent role in behavioral execution processes than in direct cognitive–affective associations with sleep outcomes. Overall, these findings support a process-oriented account of sleep difficulties in the digital context, highlighting the joint roles of cognitive–affective vulnerabilities and behavioral execution mechanisms in shaping sleep outcomes. Within this framework, FoMO-related influences on sleep appear to be more clearly expressed when translated into sustained smartphone engagement, particularly among individuals with higher sensation seeking. The present study contributes to a more nuanced understanding of digital media-related sleep problems and provides implications for developing mechanism-based interventions in higher education settings.

## Figures and Tables

**Table 1:** Descriptive statistics and interrelations among all of the observed variables.

Variable	*M*	*SD*	1	2	3	4	5
1. Age	19.65	1.20	1				
2. FoMO	2.10	0.52	−0.01	1			
3. PSU	2.62	0.62	−0.02	0.37 **	1		
4. SS	3.30	0.89	0.02	0.23 **	0.15 **	1	
5. SQ	10.27	2.10	0.05	0.26 **	0.31 **	0.09 **	1

N = 1124 FoMO Fear of Missing Out, PSU Problematic Smartphone Use, SS Sensation Seeking, SQ Sleep Quality. ** *p* < 0.01.

**Table 2:** The mediation analysis of problematic smartphone use.

Variable	Model 1 (SQ)			Model 2 (PSU)				Model 3 (SQ)	
	*β*	*t*	95% *CI*	*β*	*t*	95% *CI*	*β*	*t*	95% *CI*
Gender	−0.19	−3.19 **	[−0.30, −0.07]	−0.24	−4.26 ***	[−0.35, −0.13]	−0.13	−2.26 *	[−0.24, −0.02]
Age	0.05	2.16 *	[0.005, 0.10]	−0.001	−0.04	[−0.05, 0.04]	0.05	2.22 *	[0.01, 0.10]
FoMO	0.27	9.22 ***	[0.21, 0.32]	0.38	13.82 ***	[0.33, 0.44]	0.18	5.78 ***	[0.12, 0.24]
PSU							0.24	7.76 ***	[0.18, 0.30]
*R*		0.28			0.39			0.35	
*R* ^2^		0.08			0.15			0.12	
*F*		30.96 ***			66.56 ***			39.52 ***	

N = 1124 FoMO Fear of Missing Out, PSU Problematic Smartphone Use, SQ Sleep Quality. * *p* < 0.05, ** *p* < 0.01, *** *p* < 0.001.

**Table 3:** The analyses of total, direct, and indirect effects.

	Effects	Boot SE	Boot LLCI	Boot ULCI
Total effect	0.27	0.03	0.21	0.32
Direct effect	0.18	0.03	0.12	0.24
Indirect effect	0.09	0.01	0.06	0.12

N = 1124 Boot strap sample size = 5000. LL low limit, CI confidence interval, UL upper limit.

**Table 4:** The moderated mediation model.

Variable	Mediator Variable Model (PSU)			Dependent Variable Model (SQ)		
	*β*	*t*	95% *CI*	*β*	*t*	95% *CI*
Gender	−0.24	−4.32 ***	[−0.35, −0.13]	−0.13	−2.30 *	[−0.24, −0.02]
Age	−0.001	−0.04	[−0.05, 0.04]	0.05	2.28 *	[0.01, 0.10]
FoMO	0.35	11.95 ***	[0.29, 0.41]	0.15	4.88 ***	[0.09, 0.22]
SS	0.07	2.55 *	[0.02, 0.13]	0.02	0.66	[−0.04, 0.08]
FoMO × SS	0.06	2.44 *	[0.01, 0.12]	0.05	1.62	[−0.01, 0.11]
PSU × SS				0.06	2.10 *	[0.003, 0.11]
PSU				0.22	7.37 ***	[0.16, 0.28]
*R*		0.40			0.36	
*R* ^2^		0.16			0.13	
*F*		42.66 ***			24.47 ***	

N = 1124 FoMO Fear of Missing Out, PSU Problematic Smartphone Use, SS Sensation Seeking, SQ Sleep Quality * *p* < 0.05, *** *p* < 0.001.

**Table 5:** The conditional effects at different levels of sensation seeking.

	SS	Conditional Effects	SE	LLCI	ULCI
FoMO → PSU	*M* − 1*SD*	0.28	0.05	0.19	0.37
	*M*	0.35	0.03	0.29	0.41
	*M* + 1*SD*	0.40	0.03	0.34	0.46
PSU → SQ	*M* − 1*SD*	0.16	0.04	0.08	0.25
	*M*	0.23	0.03	0.17	0.29
	*M* + 1*SD*	0.27	0.04	0.20	0.34
Indirect effect (FoMO → PSU → SQ)	*M* − 1*SD*	0.05	0.02	0.02	0.08
	*M*	0.08	0.01	0.06	0.11
	*M* + 1*SD*	0.11	0.02	0.08	0.14

N = 1124 FoMO Fear of Missing Out, PSU Problematic Smartphone Use, SS Sensation Seeking, SQ Sleep Quality Bootstrap confidence intervals were used for indirect effects.

## Data Availability

The data that support the findings of this study are available from the corresponding author upon reasonable request.
